# Effects of Non-Face-to-Face Chronic Care Management on Service Utilization and Outcomes Among US Medicare Beneficiaries with Diabetes

**DOI:** 10.1007/s11606-024-08667-0

**Published:** 2024-02-21

**Authors:** Dongzhe Hong, Charles Stoecker, Yixue Shao, Elizabeth Nauman, Vivian Fonseca, Gang Hu, Alessandra N. Bazzano, Edmond K. Kabagambe, Lizheng Shi

**Affiliations:** 1 Department of Health Policy and Management, School of Public Health and Tropical Medicine, Tulane University, New Orleans, LA USA; 2 Program On Regulation, Therapeutics, and LAW (PORTAL), Division of Pharmacoepidemiology and Pharmacoeconomics, Department of Medicine, Brigham and Women’s Hospital, Boston, MA USA; 3 Harvard Medical School, Boston, MA USA; 4 Louisiana Public Health Institute, New Orleans, LA USA; 5 Section of Endocrinology, Tulane University Health Sciences Center, New Orleans, LA USA; 6 Pennington Biomedical Research Center, Baton Rouge, LA USA; 7 Department of Social, Behavioral, and Population Sciences, School of Public Health and Tropical Medicine, Tulane University, New Orleans, LA USA; 8 Division of Academics, Ochsner Center for Outcomes Research, Ochsner Health, New Orleans, LA USA; 9 Penn Medicine Lancaster General Health, Lancaster, PA USA

## Abstract

**Background:**

Type 2 diabetes mellitus (T2DM) results in heavy economic and disease burdens in Louisiana. The Centers for Medicare and Medicaid Services has reimbursed non-face-to-face chronic care management (NFFCCM) for patients with two or more chronic conditions since 2015.

**Objective:**

To assess the impacts of NFFCCM on healthcare utilization and health outcomes.

**Design, Setting, and Participants:**

This retrospective cohort study included Medicare fee-for-service beneficiaries with T2DM and at least one additional chronic disease between 2014 and 2018.

**Exposures:**

At least one record of NFFCCM Current Procedural Terminology codes.

**Main Measures:**

The health outcomes in the study included major adverse cardiovascular events (MACE), all-cause mortality, and heart failure. The monthly service utilization and continuity of care index for primary care were also included. The propensity score method was used to balance the baseline differences between the two groups. Weighted multivariate regression models were developed using propensity score weights to assess the impacts of NFFCCM on outcomes.

**Key Results:**

During the 5 years of study period, 8415 patients among the 118,643 Medicare beneficiaries received at least one NFFCCM. Patients receiving any NFFCCM had reduced healthcare utilization compared with patients not receiving NFFCCM, including 0.012 (95% CI − 0.014 to − 0.011; *p* < 0.001) fewer monthly hospital admissions, 0.017 (95% CI − 0.019 to − 0.016; *p* < 0.001) fewer monthly ED visits, and 0.399 (95% CI 0.375 to 0.423; *p* < 0.001) more monthly outpatient encounters. Patients receiving NFFCCM services had lower MACE event rates of 7.4% (95% CI 7.1 to 7.8%; *p* < 0.001), all-cause mortality rate of 7.8% (95% CI 7.4 to 8.1%; *p* < 0.001), and heart failure rate of 0.3% (95% CI 0.2 to 0.5%; *p* < 0.001), respectively.

**Conclusions and Relevance:**

These findings suggest that reimbursement for NFFCCM was associated with the shifting high-cost utilization to lower-cost primary health care settings among patients with diabetes in Louisiana.

## BACKGROUND

Type 2 diabetes mellitus (T2DM) is one of the most prevalent chronic diseases in the world, and results in heavy economic and disease burdens, both within the US healthcare system and globally.^1–3^ The prevalence of diabetes in Americans aged ≥ 20 years increased from 6.6% in 1976–1980 to 14.3% in 2017–2018.^4–6^ The majority of the costs associated with T2DM are attributed to micro- and macrovascular complications, such as retinopathy, nephropathy, neuropathy, coronary heart disease (CHD), and stroke.^7–9^ People with T2DM have 2–4 times greater risk for future cardiovascular disease (CVD) compared to people without diabetes.^10–12^ CVD is the leading cause of morbidity and mortality and accounts for $37.3 billion in cardiovascular-related spending per year among patients with T2DM.^13^ According to the Behavioral Risk Factor Surveillance System from CDC, Louisiana has the 4th highest prevalence of diagnosed diabetes (14.1%), and the 10th highest rate of CVD (5.0%) and among all the states in 2020.^14^

Elderly patients with diabetes covered by Medicare usually have multiple chronic conditions including diabetes.^15^ In order to improve chronic care management among elderly people with multiple chronic conditions, a new policy went into effect on January 1, 2015, under which non-face-to-face chronic care management (NFFCCM) is reimbursable by the Centers for Medicare and Medicaid Services (CMS). The Current Procedural Terminology (CPT) code 99,490 for NFFCCM reimbursement went into effect in 2015. Codes 99,487 and 99,489 went into effect in 2017 to provide additional reimbursement for more time spent delivering NFFCCM services. ^16,17^

Although several studies found chronic care management (CCM) was associated with lower health care utilization, spending, and mortality for both Medicare and Medicaid beneficiaries with complex chronic conditions, ^18–23^ only limited studies have assessed the impact of CMS’s newer reimbursement strategy for NFFCCM.^24–27^ Specifically, Schurrer et al. found that receipt of NFFCCM coded with 99,490 decreased the likelihood of admission for common chronic conditions and increased primary care utilization using Medicare claims.^27^ Thus, our study assessed the impacts of NFFCCM on healthcare utilization and health outcomes among Medicare beneficiaries with T2DM in Louisiana.

## METHODS

### Data and Study Population

The study obtained the Medicare fee-for-service (FFS) claims for Louisiana beneficiaries from CMS, for 5 years between January 1, 2014, and December 31, 2018. The data included Medicare FFS claims submitted by professional providers (e.g., physicians, physician assistants, clinical social workers, nurse practitioners, institutional outpatient providers), and inpatient hospital providers.

We used the CMS Conditions Data Warehouse (CCW) condition algorithms to identify chronic conditions in our study.^28^ There are 27 chronic condition categories in the algorithms, including diabetes, heart failure, and stroke. Type 2 diabetes was defined as at least one inpatient or two outpatient claims with the International Classification of Diseases, Ninth/Tenth Revision, Clinical Modification (ICD 9/10-CM) codes forT2DM. In our study, cardiovascular disease includes CHD, heart failure, and stroke, which were identified as at least one inpatient or outpatient claim with ICD 9/10-CM codes of the corresponding diagnoses.

**Figure 1:**
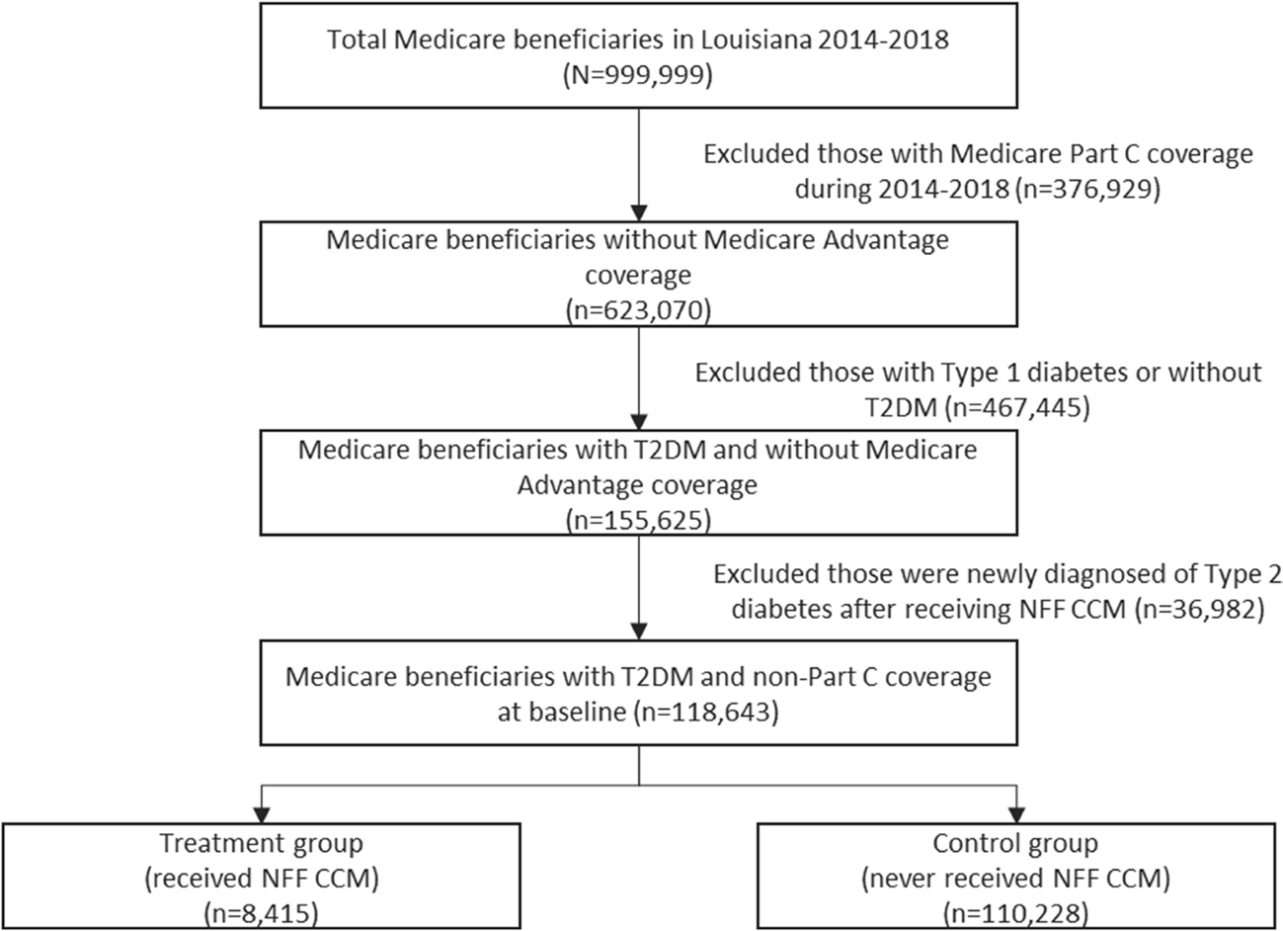
Louisiana Medicare population with T2DM selection.

Figure 1 presents a flow diagram of patient selection. Among 999,999 Medicare beneficiaries obtained from the Medicare fee-for-service claims data in Louisiana from 2014 to 2018, 376,929 beneficiaries were excluded since they had ever enrolled in Medicare advantage plan (Medicare Part C coverage) during the study period and their claims information may be incomplete since our data source is Medicare fee-for-service claims. Another 467,445 beneficiaries were excluded since they had type 1 diabetes or they did not have T2DM during the study period, and 36,982 patients were excluded since they had T2DM after the initiation date of receiving NFFCCM services. Therefore, the final analytical sample had 118,643 Medicare beneficiaries with T2DM and Medicare Part A and Part B coverage, including 8415 beneficiaries in the treatment group who received at least one NFFCCM service and 110,228 beneficiaries in the control group.

### Study Design

The intervention group comprised beneficiaries who received NFFCCM under three billing codes (99,490, 99,487, and/or 99,489) in Medicare claims, and the dates of the first NFFCCM coded were the initiation dates. In the control group, initiation dates were randomly assigned for untreated beneficiaries based on the distribution of initiation dates in the treated population. The baseline period was defined as 12 months before the initiation date of NFFCCM. All covariates, including demographics, chronic conditions, and healthcare utilization, were identified during the baseline period. The follow-up period was defined as the time period from initiation date to beneficiary’s death or the last date of Medicare enrollment or 12/31/2018. Outcomes were identified during the follow-up.

### Measurement of Outcomes

#### Health Outcomes

Health outcomes in this study included (1) major adverse cardiovascular events (MACE), including all-cause mortality, myocardial infarction (MI), and stroke, which were measured as binary variables indicating whether they were identified during the follow-up period.^29–35^ ICD-9-CM and ICD-10-CM codes were used to identify MI (ICD-9-CM code 410, ICD-10-CM code I21) and stroke (ICD-9-CM codes 430–434, 436, ICD-10-CM codes I60–I66); (2) all-cause mortality; (3) hospitalization for heart failure was identified by ICD-9-CM codes 402.11, 402.01, 402.91, and 428, and ICD-10-CM code I50 in inpatient (IP) settings.

#### Healthcare Utilization

Healthcare utilization measures included the number of hospital admissions, emergency department (ED) visits, and outpatient (OP) visits per month. The number of hospital admissions was identified as the distinct admission and discharge date in inpatient claims. The ED claims in the Medicare FFS Outpatient and Inpatient files are identified via Revenue Center Code values of 0450–0459 (emergency room) or 0981 (professional fees-emergency room).^36^ The number of OP visits was identified as the distinct date of service in OP settings. Besides the frequency of healthcare utilization, we also assessed the continuity of care (COC) for primary care. We used the Bice-Boxerman continuity of care index (COCI) to identify the density of OP services to a patient provided by the same physician.^37–40^ In our study, we counted primary care provider (PCP) visits, including general practitioners (CMS specialty code 01), family practitioners (CMS specialty code 08), internal medicine specialists without subspecialty training (CMS specialty code 11), physician assistants (CMS specialty code 97), and nurse practitioners (CMS specialty code 50). The COC index values range from zero (each visit made to a different provider) to one (all visits made to a single provider).^38^

### Statistical Analyses

The study compared outcomes in Medicare beneficiaries who received NFFCCM services and those who did not based on the presence or absence of reimbursement codes for NFFCCM. Propensity score weighting was used to balance baseline differences between the two groups. To find a group of beneficiaries resembling NFFCCM beneficiaries, group-based trajectory modeling was used to identify several subgroups who shared a similar trend of outpatient visits based on the Bayesian information criterion, and each trajectory group should have more than 5% of the population contributing to it.^41–44^ The indicators of trajectory groups were included as covariates in the propensity score weighting process to obtain a successful balance between treatment and control groups. The standardized mean difference (SMD) was used to measure the balance after propensity score weighting.

Multiple weighted linear or logistic regression models were developed using propensity score weights to assess the impact of NFFCCM on each outcome. To address the concerns about health systems selecting patients based on their likelihood of better survival, we conducted sensitivity analysis to assess the all-cause mortality in different time periods of follow-up. Multiple comparisons were adjusted by the conservative Bonferroni correction to the standard alpha-value of 0.05 by the number of comparisons.^45^ All analyses were performed using SAS 9.4 and Stata 15.1. A STATA plugin for package group-based trajectory balancing was used in the analyses.^46^

## RESULTS

Table 1 describes the baseline characteristics of the 118,643 Medicare beneficiaries. Group-based trajectory balancing and propensity score weighting methods were used to obtain comparable characteristics between the treatment (*n* = 8415) and control (*n* = 110,228) groups. All covariates were balanced between the treatment group and control group after propensity score weighting as the standardized mean differences between the two groups were all less than 0.1. For both groups, the mean age at first NFFCCM services was about 73 years old, 57.9% of both groups were females, nearly 28% were Black, and 2.1% were Hispanic. The most prevalent chronic condition among the study population was hypertension (98.2%). Other prevalent chronic conditions at baseline included arthritis and hyperlipidemia. In addition, 57.3% of the treatment group and 57.1% of the control group had CHD, while 46.8% of both treatment and control groups had MACE at baseline. The patients in the analytical sample had an average of 39.9 months of data before the initiation date of receiving NFFCCM services. The mean of monthly hospital admissions was 40 per 1000 patients, the mean of monthly ED visits was 80 per 1000 patients, and the mean of monthly outpatient visits was 255 per 1000 patients at baseline. The continuity of care index (COCI) for primary care providers was 0.46.

**Table 1:** Baseline Characteristics Before and After Propensity Score Weighting For Patients with Type 2 Diabetes

	Unweighted			Weighted		
Variables	Treatment (*n* = 8415)	Control (*n* = 110,228)	SMD	Treatment (*n* = 8415)	Control (*n* = 110,228)	SMD
Demographics						
Age at first NFFCCM, years, *n* (years)	73.18	73.37	2.00%	73.18	73.16	0.00%
Age 70–80 at first NFFCCM, %	41.90%	39.50%	− 5.10%	41.90%	41.90%	− 0.20%
Age 80–85 at first NFFCCM, %	13.90%	13.20%	− 1.80%	13.90%	13.90%	0.00%
Age > 85 at first NFFCCM, %	10.60%	13.70%	9.50%	10.60%	10.60%	0.00%
Female, %	57.90%	54.70%	− 6.50%	57.90%	57.90%	− 0.10%
Race/ethnicity, %						
Black	28.20%	29.00%	1.90%	28.20%	28.10%	− 0.20%
Asian	1.10%	1.10%	− 0.70%	1.10%	1.20%	0.40%
Others	0.50%	0.60%	1.10%	0.50%	0.50%	0.20%
Hispanic	2.10%	2.00%	− 1.00%	2.10%	2.10%	0.10%
Chronic conditions, %						
Hypertension	98.20%	96.60%	− 10.00%	98.20%	98.20%	− 0.10%
Alzheimer’s disease	3.90%	6.40%	11.70%	3.90%	3.90%	0.20%
Arthritis	80.50%	74.60%	− 14.20%	80.50%	80.40%	− 0.10%
Asthma	19.00%	14.00%	− 13.30%	19.00%	18.90%	− 0.30%
Atrial fibrillation	24.40%	21.60%	− 6.70%	24.40%	24.40%	− 0.10%
Autism	0.10%	0.10%	1.90%	0.10%	0.10%	0.00%
Cancer	24.80%	24.20%	− 1.40%	24.80%	24.90%	0.30%
Chronic obstructive pulmonary disease	45.10%	38.90%	− 12.50%	45.10%	44.90%	− 0.30%
Chronic kidney disease	35.00%	32.80%	− 4.60%	35.00%	34.90%	− 0.20%
Depression	35.30%	33.20%	− 4.50%	35.30%	35.20%	− 0.20%
Heart failure	36.00%	29.50%	− 13.90%	36.00%	35.80%	− 0.50%
Hyperlipidemia	83.40%	80.50%	− 7.80%	83.40%	83.30%	− 0.30%
Coronary heart disease	57.30%	48.80%	− 17.10%	57.30%	57.10%	− 0.30%
Osteoporosis	13.30%	9.50%	− 12.20%	13.30%	13.30%	0.10%
Stroke	42.70%	36.30%	− 12.90%	42.70%	42.60%	0.00%
Acute MI	10.90%	10.30%	− 1.80%	10.90%	10.90%	0.00%
MACE	46.80%	41.10%	− 11.50%	46.80%	46.80%	− 0.10%
Other variables						
Months in sample before initiation date, *n*	39.92	38.02	− 15.00%	39.92	39.93	0.00%
Mean hospital admissions per month before initiation date, *n*	0.04	0.04	7.00%	0.04	0.04	1.00%
Mean ED visits per month before initiation date, *n*	0.08	0.09	2.00%	0.08	0.08	0.00%
Mean outpatient visits per month before initiation date, *n*	2.55	2.63	3.00%	2.55	2.55	0.00%
Continuity of Care Index, *n*	0.46	0.46	− 1.00%	0.46	0.46	0.00%
Trajectory group 1, %	56.80%	62.60%	11.80%	56.80%	56.90%	0.30%
Trajectory group 2, %	37.80%	28.70%	− 19.40%	37.80%	37.60%	− 0.30%
Trajectory group 3, %	5.40%	8.70%	12.90%	5.40%	5.50%	0.20%

*SMD*, standardized mean difference; *NFFCCM*, non-face-to-face chronic care management; *MI*, myocardial infarction; *MACE*, major adverse cardiovascular events; *ED*, emergency department

Patients receiving any NFFCCM had reduced healthcare utilization compared with patients not receiving NFFCCM, including 0.012 (95% CI − 0.014 to − 0.011; *p* < 0.001) fewer monthly hospital admissions, 0.017 (95% CI − 0.019 to − 0.016; *p* < 0.001) fewer monthly ED visits, and 0.399 (95% CI 0.375 to 0.423; *p* < 0.001) more monthly outpatient encounters. Patients receiving NFFCCM services had lower MACE event rates of 7.4% (95% CI 7.1 to 7.8%; *p* < 0.001) all-cause mortality rate of 7.8% (95% CI 7.4 to 8.1%; *p* < 0.001), acute myocardial infarction (MI) event rate of 0.3% (95% CI 0.1 to 0.4%; *p* < 0.001), and event rate of hospitalization for heart failure of 0.3% (95% CI 0.2 to 0.5%; *p* < 0.001), respectively.

Table 2 presents that patients receiving any NFFCCM had reduced healthcare utilization compared with patients not receiving NFFCCM. The mean (SD) duration of follow-up was 1.46 (1.01) years. Compared with those without the NFFCCM services, patients receiving any NFFCCM had 0.012 (95% CI − 0.014 to − 0.011; *p* < 0.001) fewer monthly hospital admissions or 12 fewer hospital admissions out of 1000 patients, 0.017 (95% CI − 0.019 to − 0.016; *p* < 0.001) fewer monthly ED visits or 17 fewer monthly ED visits out of 1000 patients, and 0.399 (95% CI 0.375 to 0.423; *p* < 0.001) more monthly outpatient visits or 399 more outpatient visits out of 1000 patients. Receiving any NFFCCM was not associated with monthly outpatient utilization when NFFCCM encounters were excluded. In addition, receiving any NFFCCM was associated with a statistically significant higher COCI for primary care physicians of 0.094 (95%CI 0.091 to 0.097; *p* < 0.001). Furthermore, the four measures of health care utilization were significant at the *p* < 0.001 level, passing the adjusted Bonferroni correction for *p*-value of 0.01.

**Table 2:** Impacts of Receiving Any NFFCCM Services on Health Care Utilization

Outcomes	Hospital admissions per month	ED visits per month	OP visits per month	OP visits per month (excluding the NFFCCM encounters)	COCI
Treatment effects coefficients	− 0.012^***^	− 0.017^***^	0.399^***^	− 0.001	0.094^***^
95% confidence interval	[− 0.014, − 0.011]	[− 0.019, − 0.016]	[0.375, 0.423]	[− 0.025, 0.023]	[0.091, 0.097]
*p*-value	< 0.001	< 0.001	< 0.001	0.934	< 0.001
*N* treatment	8341	8341	8341	8341	8142
*N* control	106,632	106,632	106,632	106,632	94,066
*N* total	114,973	114,973	114,973	114,973	102,208

*p < 0.05, **p < 0.01, ***p < 0.001. The number of inpatient visits per month = total number of inpatient visits / the number of corresponding follow-up months. We controlled for (1) demographic characteristics, including age, sex, race, and ethnicity; (2) chronic disease status, including hypertension, Alzheimer’s disease, arthritis, asthma, atrial fib, autism, cancer, COPD, ESRD, depression, heart failure, hyperlipidemia, chronic heart disease, osteoporosis, and stroke; (3) other outcomes before initiation date; and (4) outpatient trajectory balancing groups’ score, including three outpatient trajectories. Applying the conservative Bonferroni correction, the significant *p*-values are below 0.05/5 = 0.01. Coefficients are followed by 95% CI and *p*-value. The mean (SD) duration of follow-up was 1.46 (1.01) years

Table 3 presents the impacts of receiving any NFFCCM on five health outcomes with the adjusted Bonferroni correction of *p*-value of 0.01. Compared with those without the NFFCCM services, patients receiving the NFFCCM services had lower MACE event rate of 7.4% (95% CI 7.1 to 7.8%; *p* < 0.001). Patients receiving NFFCCM services also had lower all-cause mortality rate of 7.8% (95% CI 7.4 to 8.1%; *p* < 0.001), lower acute myocardial infarction (MI) event rate of 0.3% (95% CI 0.1 to 0.4%; *p* < 0.001), and lower event rate of hospitalization for heart failure of 0.3% (95% CI 0.2 to 0.5%; *p* < 0.001), respectively. Receiving NFFCCM services was not associated with changes/differences in rates of stroke. The four measures of health outcomes were significant at the *p* < 0.001 level, passing the adjusted Bonferroni correction for *p*-value of 0.01.

**Table 3:** Impacts of Receiving Any NFFCCM Services on Health Outcomes

Outcomes	MACE	All-cause mortality	Acute MI	Stroke	Heart failure
Treatment effects coefficients (SE)	− 0.074^***^	− 0.078^***^	− 0.003^***^	0.001	− 0.003^***^
95% confidence interval	[− 0.078, − 0.071]	[− 0.081, − 0.074]	[− 0.004, − 0.001]	[− 0.001, 0.003]	[− 0.005, − 0.002]
*p*-value	< 0.001	< 0.001	< 0.001	0.379	< 0.001
*N* treatment	8341	8341	8341	8341	8341
*N* control	106,632	106,632	106,632	106,632	106,632
*N* total	114,973	114,973	114,973	114,973	114,973

*p < 0.05, **p < 0.01, ***p < 0.001. The number of inpatient visits per month = total number of inpatient visits / the number of corresponding follow-up months. We controlled for (1) demographic characteristics, including age, sex, race, and ethnicity; (2) chronic disease status, including hypertension, Alzheimer’s disease, arthritis, asthma, atrial fib, autism, cancer, COPD, ESRD, depression, heart failure, hyperlipidemia, chronic heart disease, osteoporosis, and stroke; (3) other outcomes before initiation date; and (4) outpatient trajectory balancing groups’ score, including three outpatient trajectories. Applying the conservative Bonferroni correction, the significant *p*-values are below 0.05/5 = 0.01. Coefficients are followed by 95% CI and *p*-value. The mean (SD) duration of follow-up was 1.46 (1.01) years

Table 4 presents the impacts of receiving any NFFCCM on all-cause mortality in different time periods of follow-up as sensitivity analyses. Receiving any NFFCCM decreased the probability of all-cause mortality across all the time periods of follow-up. Specifically, there was a 2.4% (95% CI 2.3 to 2.6%; *p* < 0.001) decrease in all-cause mortality within 0–3 months after the index date of NFFCCM services. There was a 2.6% (95% CI 2.4 to 2.8%; *p* < 0.001) decrease in all-cause mortality 3–6 months after the index date. In the 6–12 months and 12 + months after initiating NFFCCM, there were 5.0% (95% CI 4.7 to 5.3%; *p* < 0.001) and 3.9% (95% CI 3.5 to 4.3%; *p* < 0.001) decreases, respectively, in all-cause mortality.

**Table 4:** Impacts of Receiving Any NFFCCM on All-Cause Mortality in Different Time Periods of Follow-up

Follow-up times after first NFFCCM before death	0–3 months	3–6 months	6–12 months	> 12 months
Treatment effects coefficients (SE)	− 0.024^***^	− 0.026^***^	− 0.050^***^	− 0.039^***^
95% confidence interval	[− 0.026, − 0.023]	[− 0.028, − 0.024]	[− 0.053, − 0.047]	[− 0.043, 0.035]
*p*-value	< 0.001	< 0.001	< 0.001	< 0.001
*N* treatment	7955	7149	5612	5208
*N* control	102,289	90,038	70,055	61,824
*N* total	110,244	97,187	75,667	67,032

*p < 0.05, **p < 0.01, ***p < 0.001. The number of inpatient visits per month = total number of inpatient visits / the number of corresponding follow-up months. We controlled for (1) demographic characteristics, including age, sex, race, and ethnicity; (2) chronic disease status, including hypertension, Alzheimer’s disease, arthritis, asthma, atrial fib, autism, cancer, COPD, ESRD, depression, heart failure, hyperlipidemia, chronic heart disease, osteoporosis, and stroke; (3) other outcomes before initiation date; and (4) outpatient trajectory balancing groups’ score, including three outpatient trajectories. Coefficients are followed by 95% CI and *p*-value. The mean (SD) duration of follow-up was 1.46 (1.01) years

## DISCUSSION

This study of the impact of reimbursement for NFFCCM services found more outpatient visits and fewer inpatient admissions and ED visits among patients with T2DM. Our core estimates indicate a decrease of 14.4 (0.012 × 12 × 100) inpatient admissions per 100 patients annually and a decrease of 20.4 (0.017 × 12 × 100) ED visits per 100 patients annually, which provides strong evidence to encourage the use of NFFCCM in diabetes care. Further, the increase in outpatient use in the NFFCCM group is due to the NFFCCM services, while all the other types of outpatient visits remain stable.

Our findings from Medicare claims showed a similar impact of NFFCCM on health care utilization as a CMS-funded study among Medicare beneficiaries.^27^ The study found reduced rates of ED visits and hospitalizations among the NFFCCM beneficiaries. But the previous findings were limited to only non-complex NFFCCM before the complex NFFCCM services were available for Medicare beneficiaries with at least two chronic conditions. Our findings further demonstrate the positive effects of NFFCCM, including both non-complex NFFCCM and complex NFFCCM, on decreasing hospitalization for diabetes patients with any other chronic diseases.

In addition, our results further demonstrate the impact of NFFCCM on health outcomes in diabetes. We found significantly lower cardiovascular event rates, while a risk reduction of 0.3% for both acute MI and heart failure may not be clinically meaningful, which were smaller than the treatment effects of newer diabetes medications, such as dapagliflozin observed in the clinical trial with a lower rate of 1.1%.^47^ The NFFCCM services include structured recording of patient health information, 24/7 access to providers, and comprehensive care management, which are free of side effects and may be more cost-effective and acceptable to patients.^17^ The reduction of MACE and mortality rates could be associated with the better control of blood glucose, blood pressure, and low-density lipoprotein outcomes.^48^Further research may benefit from assessing cardiovascular death or including the reason of death. The combination of these findings could be used to support reimbursement of NFFCCM services, and we provide evidence for practitioners and patients to utilize NFFCCM in diabetes care. Policymakers could take into consideration covering more NFFCCM services or other remote services of CCM in the future.

There is an important issue of whether the NFFCCM services were additions to other outpatient care or substitutes because of its implications for cost and administrative complexity. We found there were no significant increases in all the other outpatient visits in Medicare claims analysis. Our finding based on claims records suggested that the NFFCCM services were additive to relatively constant outpatient visits, indicating that NFFCCM participation may be a marker of patient commitment to self-care.

Our study also found an improvement in continuity of primary care, which is often considered a core value of patient care in primary care medicine.^49^ The increased COCI suggests that NFFCCM beneficiaries may have been encouraged to see the same primary care physician over time, which may have improved health outcomes as a result of having one person managing their healthcare needs.^49^

This study has several limitations. The major limitation of this study is that we do not have random assignment of NFFCCM. Selection into treatment may be initiated by clinicians that think patients may benefit from NFFCCM either because of the severity of their disease or likelihood of complying to the intervention and thus potentially introduce confounding by indication. If this clinical judgment is based on unobservable characteristics in our study, we may have bias in our estimates. Our study might be influenced by self-selection bias, where patients already committed to self-care are more likely to participate, then the results would overestimate the impacts of NFFCCM. To mitigate potential bias from lack of randomization, we used trajectory balancing and propensity score weighting to balance the NFFCCM and the control groups on measured variables, while unmeasured confounders could still lead to biased results. The patient population created by the propensity score weighting was different from Medicare population, including having overall more chronic conditions, more women, fewer individuals over 85 years, and less Alzheimer’s disease, which would yield the generalizability to the broader Medicare population. Additionally, we lack physician and organizational level factors and other socioeconomic factors, such as education and income, to address potential confounding.

Second, Medicare claims data does not have clinical biomarkers that reflect the impacts of NFFCCM on glycemic control and other important biomarkers in diabetes care. Thus, the direct effects of NFFCCM on some clinical outcomes cannot be analyzed in this study due to the data limitations. Future study could improve the data completeness by using a linked Medicare FFS claims to EHR. In our study’s data, we did not have hospice information to better adjust for patients’ general condition, which is a possible mechanism contributing to the high mortality reduction. We assessed the impacts of NFFCCM on all-cause mortality at different follow-up times in order to address this issue, and found significant reductions in mortality in 6–12 months, as well as > 12 months after excluding those who died in 0–6 months, who might have used hospice services. We used logistic regression to assess mortality incidence as the relatively short follow-up duration might not be critical for a non-medication intervention; however, future studies would benefit by applying survival analyses with longer follow-up data. These factors limit the generalizability of our results, and stronger evidence of the impact of NFFCCM may be found in a larger database with more NFFCCM recipients and clinical measurements.

## CONCLUSIONS

Our study is the first real-world study using Medicare claims data to present evidence that the reimbursement of NFFCCM, complex or non-complex, was associated with the shifting high-cost health utilization to low-cost primary health care setting among patients with diabetes in Louisiana. Policymakers could consider covering more NFFCCM services or other remote services of CCM in the future. Incorporating modern technology for disease management could be coupled with NFFCCM to provide significant clinical benefits.

## Data Availability

The datasets generated during and/or analyzed during the current study are not publicly available due to the CMS Research Identifiable File Data Use Agreement, which prohibits the sharing of raw data.
